# Retraction: LncRNA ANRIL affects the sensitivity of ovarian cancer to cisplatin via regulation of let-7a/HMGA2 axis

**DOI:** 10.1042/BSR-2018-2101_RET

**Published:** 2024-07-31

**Keywords:** ANRIL, cisplatin, HMGA2, let-7a, ovarian cancer, sensitivity

This article is being retracted from *Bioscience Reports* at the request of the Editor-in-Chief and the Editorial Board following receipt of a notification from a reader, alerting the Editorial Board to multiple similarities between data in this paper and that present in other articles previously published by different authors. Specifically:

- Samples in Figure 6A which seem to appear in Figure 7a from Deng et al, 2017 (DOI: 10.1038/srep41616), with panel rearrangement
- Figure 6B which seems to appear as Figure 7d from Deng et al, 2017 (DOI: 10.1038/srep41616), with panel rearrangement and rotation of the NC siRNA+cisplatin and ANRIL siRNA+cisplatin images
- The images in Figure 6E which seem to appear in Figure 7d-i from Ma et al, 2018 (DOI: 10.1186/s13046-018-0686-6), with contrast alterations and rotation of the ANRIL siRNA+cisplatin image.

Additionally, the Editorial Office has concerns regarding signs of editing in the Western blots of Figure 3 and Figure 5. The authors have been contacted with regards to the retraction and have not responded to the Journal's queries or the concerns raised. Given the extent of the issues raised, the Editorial Board stand by the decision to retract the article.

